# Endoscopic Treatment of Intrasheath Peroneal Tendon Subluxation

**DOI:** 10.1155/2013/274685

**Published:** 2012-12-26

**Authors:** Frederick Michels, Stéphane Jambou, Stéphane Guillo, Jan Van Der Bauwhede

**Affiliations:** ^1^Orthopaedic Department, AZ Groeninge, B. Vercruysselaan 5, 8500 Kortrijk, Belgium; ^2^Orthopaedic Department, Bordeaux Mérignac Sports Clinic, 9 rue Jean Moulin, 33700 Bordeaux, France

## Abstract

Intrasheath subluxation of the peroneal tendons within the peroneal groove is an uncommon problem. Open exploration combined with a peroneal groove-deepening procedure and retinacular reefing is the recommended treatment. This extensive lateral approach needs incision of the intact superior peroneal retinaculum and repair afterwards. We treated three patients with a painful intrasheath subluxation using an endoscopic approach. During this tendoscopy both tendons were inspected. The distal muscle fibers of the peroneus brevis tendon were resected in two patients. A partial tear was debrided in the third patient. All patients had a good result. No wound-healing problems or other complications occurred. Early return to work and sports was possible. An endoscopic approach was successful in treatment of an intrasheath subluxation of the peroneal tendons.

## 1. Introduction

Peroneal tendon dislocation is a well-known entity in literature [1]. The peroneal tendons dislocate over the lateral malleolus and cause posterolateral ankle pain and a snapping sensation. On the other hand, only a few publications exist about intrasheath peroneal subluxation, as pathology is uncommon [2, 3]. Patients with an intrasheath subluxation present with the common subjective feelings of popping, snapping, or clicking associated with pain. They do not demonstrate objective clinical evidence of subluxation over the lateral malleolus. Static and dynamic ultrasound has been shown valuable in diagnosis of this pathology [4]. It demonstrates the both peroneal tendons switching their relative positions. In the few publications intrasheath subluxation is associated with a low-lying peroneal muscle belly, a peroneus quartus muscle and tendon, a tear of 1 or both of the peroneal tendons. In all described cases the superior peroneal retinaculum was intact [2, 3].

Most patients with peroneal tendon lesions can be treated conservatively with activity modification, NSAIDs, physical therapy, footwear changes, temporary immobilization, and corticosteroid injections [5]. Operative treatment is usually reserved for patients who have failed 3 to 6 months of conservative management [6].

Surgical treatment usually uses an extensive lateral exposure to facilitate adequate visualisation, diagnosis, and treatment [7, 8]. This approach needs incision of the superior peroneal retinaculum and repair afterwards. The problems associated with long open exposures are scar formation, adhesions, and entrapment of the sural nerve [8]. Often prolonged immobilisation is needed.

In this paper, we describe an endoscopic approach in patients with intrasheath subluxation of the peroneal tendons. The hypertrophied muscle belly was resected in two patients. A partial tear of the peroneus brevis tendon was debrided in the third patient. In all patients the outcome was successful.

## 2. Case Reports

### 2.1. Case 1

A 23-year-old man had 2 years persisting pain in the right ankle despite conservative treatment consisting of activity modification, insoles, nonsteroidal anti-inflammatory medication, physical therapy, and cortisone injections. The patient was not aware of an important ankle injury. But he played soccer at regional level and had some minor ankle sprains in the years before.

Clinical examination showed localized tenderness behind the lateral malleolus. Contraction of the peroneal tendons cause a snapping movement. This snapping movement causes a reproducible painful audible click (Movie 1; see Supplementary Material available online at http://dx.doi.org/10.1155/2013/274685). The tendons were found to switch their relative positions at the level just posterior to the tip of the lateral malleolus. This was visual during clinical examination.

Dynamic ultrasonography demonstrated a switching of the both peroneal tendons. No tendon lesions were found. No dislocation out of the retromalleolar groove occurred. MRI revealed no lesions. We decided to treat this patient surgically and a tendoscopy was planned.

### 2.2. Case 2

The second patient is a 26-year-old man with complaints of retromalleolar pain during more than 6 months. He also plays soccer and has a very similar history. The diagnosis was confirmed by ultrasonography. No tendon lesions were found.

### 2.3. Case 3

The third patient is a 38-year-old man with complaints of retromalleolar pain since one year started after an ankle sprain. He is a long-distance runner, but unable to sport owing to his complaints. Ultrasonography confirmed the diagnosis of an intrasheath subluxation. MRI revealed a partial rupture of the peroneus brevis tendon.

### 2.4. Surgical Technique

The operation was performed under regional anaesthesia. The patient was placed in lateral decubitus. A tourniquet was applied.

**Figure 1:**
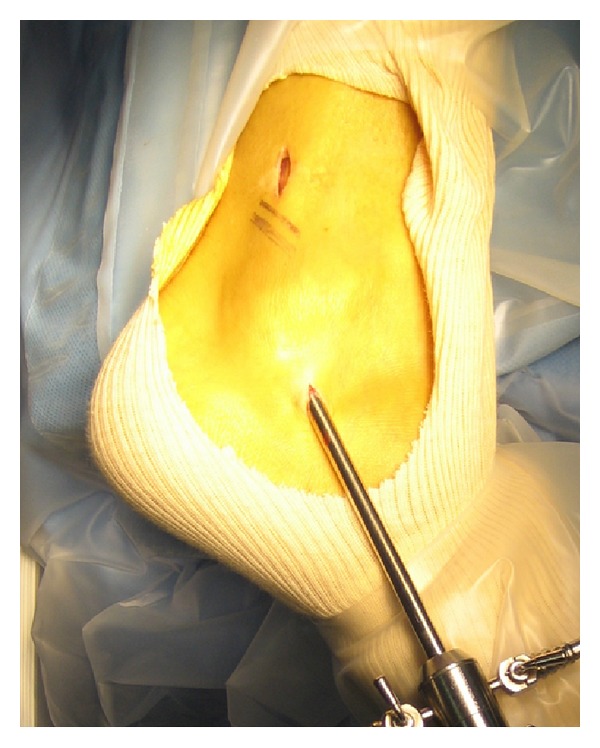
Position of endoscopic portals.

**Figure 2:**
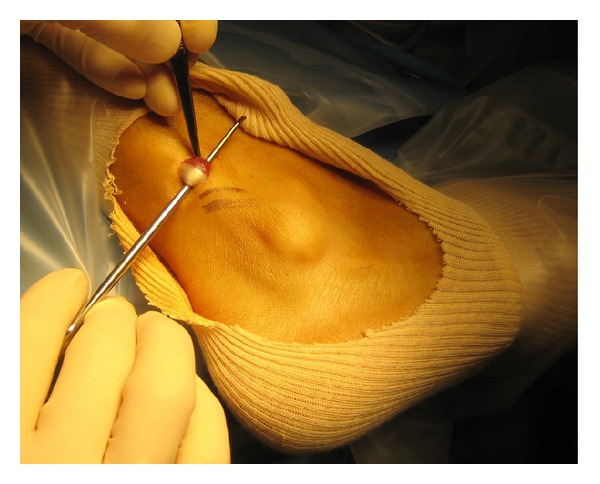
Distal location of muscle fibres around peroneus brevis tendon.

**Figure 3:**
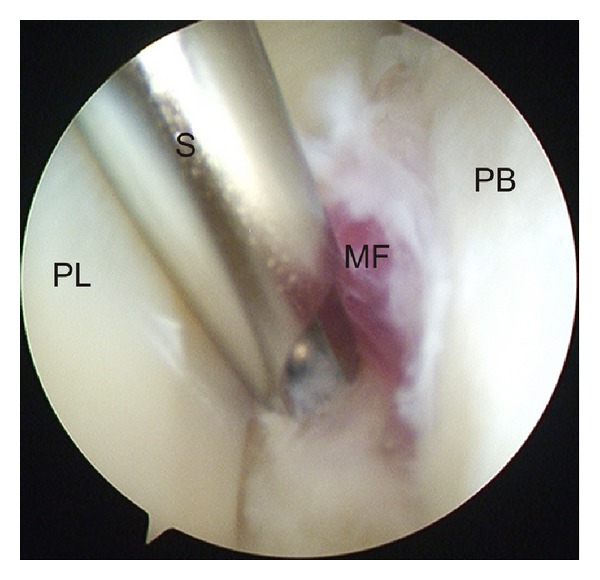
Endoscopic view of resection of distal muscle fibers. (PL: peroneus longus tendon, S: shaver, MF: muscle fibers, PB: peroneus brevis tendon).

Two portals were performed (Figure 1). The proximal portal is made first. An incision is made 2.5 cm above the tip of the lateral malleolus. The tendon sheath is incised and a blunt trocar is introduced. The cannula is aimed distally along the course of the tendons. We use a 4 mm arthroscope with an inclination of 30°. After filling the tendon sheath with a saline solution, the second portal is performed by use of transillumination. The distal portal is situated 2.0 cm distal to the fibula tip. A blunt probe was introduced. The full length of both tendons was probed. In the first two patients no tendon ruptures were found. The retinaculum was intact. No abnormalities of the peroneal groove were found. The most remarkable finding was the very distal location of muscle fibers around the peroneus brevis tendon (Figure 2). With a shaver the distal muscle fibers were resected over a length of 2 cm (Figure 3). We used the two portals interchangeably for both the arthroscope and the resector. In the third patient a partial tear of the peroneus brevis tendon was found. The tear was debrided with the shaver.

### 2.5. Postoperative Management

Postoperative course was uneventful. Immediate mobilisation and total weight bearing was allowed. Physiotherapy was started with active and passive full range of motion exercises after 5 days. The first patient returned to sports (cycling and soccer) 2 months following surgery. The second patient returned to work after two weeks, running after 6 weeks and soccer after 10 weeks. The third patient started running after 8 weeks. Two months postoperatively all patients were pain free and no snapping sensation reoccurred. After 2 years of follow-up the patients still had a normal function and no complaints.

## 3. Discussion

The present study demonstrates the possible endoscopic treatment for intrasheath subluxation of the peroneal tendons.

The initial treatment of peroneal tendon disorders is conservatively. This nonsurgical treatment includes activity modification, physiotherapy, footwear changes, temporary immobilisation, and corticosteroid injections.

The open surgical treatment uses extensive exposure to facilitate visualisation. This is associated with scar formation and sural nerve entrapment [8].

Recently, several authors reported good to better results with an endoscopic technique. Van Dijk and Kort reported in 1998 an endoscopic approach for the diagnosis and treatment of peroneal tendon pathology [9]. Peroneal tendoscopy has been used for several indications. As a diagnosing tool it allows an extended evaluation of the tendons in a rather minimally invasive approach. It also allows to treat several disorders: adhesiolysis, synovectomy, resection of a fibular or calcaneal exostosis, debridement or suturing of partial tendon rupture, groove deepening. Lui described an endoscopic reconstruction of the peroneal retinaculum [10]. Tendoscopy has demonstrated several advantages, such as less pain, outpatient treatment, functional after-treatment, and rapid resumption of work and sport activities.

Dislocation of the peroneal tendons out of the peroneal groove is well known in literature. Only a few publications exist about intrasheath subluxations.

Raikin et al. described intrasheath subluxation of the peroneal tendons in 14 patients [2]. In thirteen patients a convex peroneal groove was found. Four patients involved a longitudinal split within the peroneus brevis tendon. Patients were treated with an open peroneal groove-deepening procedure with retinacular reefing and a surgical repair if needed. They reported good to excellent results. In our patients no groove deepening procedure was performed. According to the suggestions of Ferran and Maffulli, we do not see a convex peroneal groove as a pathologic finding [11]. The retrofibular cartilage is not formed by the concavity of the fibula, but by a relatively pronounced ridge of fibrocartilage [12]. Anatomical studies demonstrate an the incidence of a flat or convex sulcus ranging from 18 to 30% in normal cadaveric specimens [11].

Thomas et al. report 7 patients with intrasheath subluxation [3]. Six of the seven patients had either a low-lying peroneal muscle belly or a peroneus quartus muscle and tendon, 6 experienced a tear of either 1 or both peroneal tendons, and 1 of the 7 had only a peroneus brevis tendon tear without any other muscle anomaly.

In our first two cases no rupture was found. Neither was there any history of an important ankle injury. The tendon snapping was probably caused by the very distal located muscle fibers. A good result after resection of these fibers confirms this hypothesis. According to Thomas et al., the low lying peroneus muscle belly causes an increased internal cubic content of the fibro-osseous tunnel [3]. These increasing compressive forces contribute to the intrasheath subluxation. The same is true in case of an accessory peroneus muscle, a tendon tear or a convex fibular groove.

Although the number of patients in our study is low, the results are very promising. If surgery is indicated, we recommend an endoscopic approach in all patients without major tear or obvious dislocation. Dislocation of the peroneal tendons over the lateral malleolus is considered as a relative contraindication. Possible complications are prolonged inflammation, damage to the sural nerve, and thickening and scarring. These complications are more likely in an open procedure. Experience in endoscopic surgery is mandatory. Endoscopy of the peroneal tendons is a technically demanding procedure and requires the skill of an experienced arthroscopist. Concomitant lesions of the lateral ligaments should be excluded or treated.

The endoscopic procedure allows an extended investigation of the tendon in a minimally invasive approach. This approach avoids opening and suturing of the intact superior retinaculum. If no tear is found, we recommend a debridement of the distal muscle fibers. A small tear can be debrided endoscopically. In case of an unexpected major tendon tear, conversion to an open procedure is easy and always possible. As the tear is endoscopically located, the length of the incision for the open approach can be minimized.

In conclusion, tendoscopy of the peroneal tendons is a useful tool to investigate and treat peroneal tendon disorders. Endoscopic resection of the distal muscle fibers or debridement of a partial tear was successful in treatment of a persisting snapping of the peroneal tendons.

## Supplementary Material

This movie is an illustration of the clinical signs in intrasheath peroneal tendon subluxation. Clinical examination shows localized tenderness behind the lateral malleolus. Contraction of the peroneal tendons causes a snapping movement. This snapping movement causes a reproducible painful audible click. The tendons were found to switch their relative positions at the level just posterior to the tip of the lateral malleolus. This is visual during clinical examination.
